# Shared and distinct resting functional connectivity in children and adults with attention-deficit/hyperactivity disorder

**DOI:** 10.1038/s41398-020-0740-y

**Published:** 2020-02-12

**Authors:** Xiaojie Guo, Dongren Yao, Qingjiu Cao, Lu Liu, Qihua Zhao, Hui Li, Fang Huang, Yanfei Wang, Qiujin Qian, Yufeng Wang, Vince D. Calhoun, Stuart J. Johnstone, Jing Sui, Li Sun

**Affiliations:** 1 Peking University Sixth Hospital/Institute of Mental Health, 100191 Beijing, China; 2 National Clinical Research Center for Mental Disorders & Key Laboratory of Mental Health, Ministry of Health (Peking University), 100191 Beijing, China; 3 Brainnetome Center and National Laboratory of Pattern Recognition, Institute of Automation, Chinese Academy of Sciences, 100190 Beijing, China; 4 University of Chinese Academy of Sciences, Beijing, China; 5 Tri-institutional Center for Translational Research in Neuroimaging and Data Science (TReNDS) [Georgia State University, Georgia Institute of Technology, Emory University], Atlanta, GA 30303 USA; 6 Brain & Behaviour Research Institute, School of Psychology, University of Wollongong, Wollongong, Australia; 7 CAS Center for Excellence in Brain Science, Institute of Automation, Chinese Academy of Sciences, Beijing, China

**Keywords:** Diagnostic markers, ADHD, Diagnostic markers, ADHD

## Abstract

Attention-deficit/hyperactivity disorder (ADHD) often persists into adulthood, with a shift of symptoms including less hyperactivity/impulsivity and more co-morbidity of affective disorders in ADHD_adult_. Many studies have questioned the stability in diagnosing of ADHD from childhood to adulthood, and the shared and distinct aberrant functional connectivities (FCs) between ADHD_child_ and ADHD_adult_ remain unidentified. We aim to explore shared and distinct FC patterns in ADHD_child_ and ADHD_adult_, and further investigated the cross-cohort predictability using the identified FCs. After investigating the ADHD-discriminative FCs from healthy controls (HCs) in both child (34 ADHD_child_, 28 HCs) and adult (112 ADHD_adult_,77 HCs) cohorts, we identified both shared and distinct aberrant FC patterns between cohorts and their association with clinical symptoms. Moreover, the cross-cohort predictability using the identified FCs were tested. The ADHD-HC classification accuracies were 84.4% and 81.0% for children and male adults, respectively. The ADHD-discriminative FCs shared in children and adults lie in the intra-network within default mode network (DMN) and the inter-network between DMN and ventral attention network, positively correlated with total scores of ADHD symptoms. Particularly, inter-network FC between somatomotor network and dorsal attention network was uniquely impaired in ADHD_child_, positively correlated with hyperactivity index; whereas the aberrant inter-network FC between DMN and limbic network exhibited more adult-specific ADHD dysfunction. And their cross-cohort predictions were 70.4% and 75.6% between each other. This work provided imaging evidence for symptomatic changes and pathophysiological continuity in ADHD from childhood to adulthood, suggesting that FCs may serve as potential biomarkers for ADHD diagnosis.

## Introduction

Attention-deficit/hyperactivity disorder (ADHD), one of the most common neurodevelopmental disorders, is characterized by symptoms of age-inappropriate inattention, hyperactivity, and impulsivity. Relative to healthy controls (HCs), ADHD patients were significantly impaired in psychosocial, educational, and neuropsychological functioning, and the dysfunction could not be well explained by other comorbid psychopathology^1^. ADHD affects ~5% of children and adolescents, and two-thirds of children with ADHD continue to have persistent, impairing symptoms in adulthood^2^. Extant imaging studies have reported brain structural and functional differences between patients with ADHD and HCs, both in childhood and adulthood. Many functional magnetic resonance imaging (fMRI) findings heavily support that ADHD involves a distributed pattern of brain alterations^3,4^. According to Rubia et al., functional abnormalities in fronto-cortical and fronto-subcortical networks are core deficits in both children and adults with ADHD^5^.

In adults with ADHD, there seems to be a shift of symptoms including persistence of inattention, less hyperactivity/impulsivity and more co-morbidity of affective disorders^6^. Therefore, it can be inferred that except common neuro-mechanism, there may be some differences in the neural substrates of ADHD between children and adults. However, so far studies in ADHD have mostly focused on either ADHD_child_ alone or ADHD_adult_ alone. Despite extensive fMRI studies, neither common nor the age-specific underlying imaging substrates of children and adults with ADHD have been identified. What’s more, the continuity of ADHD has been challenged by recent studies^7^. Identification of shared and distinct patterns of imaging substrates in ADHD with different ages are important to further insights into the neurological mechanism of ADHD.

Lots of researches have been done over the last three decades to explore potential imaging alterations and biomarkers of ADHD. However, their findings are often inconsistent or even conflicting. In recent years, a change in perspective in etiological models of ADHD has occurred, consistent with emerging concepts in other psychiatric disorders such as schizophrenia and autism. These models shift the assumed pathological focus from regional brain abnormalities to dysfunctions in distributed network interconnectivity^8^. Recent studies have also confirmed this view. The findings of magnetic resonance diffusion tensor imaging studies indicate that white matter tract deficits in ADHD are not just confined to specific brain regions but affect the structural interconnections between regions and hence entire neural networks^9^. Similar results are shown in the largest neuroimaging meta-analysis^10^ of structural and functional experiments in children/adolescents with ADHD, including ninety-six eligible studies. The study analyzed pooled structural and functional, sub-analyses restricted to modality, and in-/decreased contrasts. No significant findings were yield in this analysis and no significant convergence was found in structural and functional regional alterations of ADHD. The overall findings highlight regional convergence is lacked in children/adolescents with ADHD, and network interactions rather than just regional abnormalities may be the underlying pathophysiology of ADHD. Although the main concept in imaging neuroscience have been assessing functional segregation in the human brain for many years, namely the localization of regionally specific functions, the pathophysiology of neuropsychiatric disorders is now being increasingly treated in a systematic perspective, i.e., function emerges from the interaction of segregated regions. Therefore, the analysis of functional connectivity (FC) is becoming more and more critical.

FC features often contains high dimensionality. Early methods such as *t*-test involved binary comparisons, which may be underpowered in identifying high-dimensional features^11^. Machine learning is known as automatically decoding regularities hidden in brain imaging data to predict disorders, which shows greater advantages in classifying psychiatric disorder characterized with deficits in heterogeneous distributed systems^12^. At present, ADHD discrimination with machine learning is mainly concerned with children^13,14^. Specially, more attention should be payed in adults with ADHD and the mutual prediction between children and adults with ADHD. Besides, high-dimensional FC matrixes with small samples as input of traditional machine learning algorithm directly would degrade the sorting performance on distinguishing ADHD from HCs. To overcome this drawback, a new feature selection method based on relative importance and ensemble learning (FS_RIEL) we proposed was used to identify both shared and age-specific FC patterns impaired in ADHD in this study.

Thus, the aim of the current study were to explore the shared and distinct FC patterns that could discriminate ADHD from HCs in both children and adults, their association with clinical symptoms, and further investigated the cross-cohort predictability using the identified FCs.

## Materials and methods

### Participants

A total of 35 children with ADHD and 28 HCs (7–14 years old) were recruited in the child dataset. The diagnosis was made by a senior psychiatrist based on the Schedule for Affective Disorders and Schizophrenia for School-Age Children-Present and Lifetime version (K-SADS-PL)^15^, a clinical and semi-structured interview based on Diagnostic and Statistical Manual of Mental Disorders-Fourth Edition (DSM-IV)^16^.

A total of 112 adults with ADHD and 77 age- and sex-matched HCs (18–40 years old) were recruited in the adult dataset. The Conner’s Adult ADHD Diagnostic Interview based on DSM-IV was completed for the diagnosis of adults with ADHD. All adult participants also underwent the Structured Clinical Interview for DSM-IV Axis I Disorders (SCID-I)^17^ by a senior psychiatrist for potential comorbidity.

All participants met the following criteria: (1) right-handed, (2) no history of head trauma with a loss of consciousness, (3) no history of neurological disorders or other severe disease, (4) no current diagnosis of major depressive disorder (MDD), schizophrenia, clinically significant panic disorder, bipolar disorder, pervasive developmental disorders, or mental retardation, (5) no excessive head movements (>3.0 mm of translation or degrees of rotation in any direction), and (6) a full-scale intelligence quotient (IQ) above 80. Furthermore, participants with any history of psychiatric disorders were also excluded as HCs.

ADHD patients were recruited from outpatient clinics of Peking University Sixth Hospital and HCs were recruited by advertisement. Adult participants were scanned in Peking University Sixth Hospital (73 ADHD, 43 HCs) and Beijing Normal University (39 ADHD, 34 HCs). All child participants were scanned in Beijing Normal University. Verbal IQ, performance IQ, and full-scale IQ were measured by the Wechsler Child/Adult Intelligence Scale, Third Edition.

The severity of inattentive symptoms, hyperactive/impulsive symptoms and total ADHD symptoms of all subjects were evaluated by the ADHD Rating Scale-IV (ADHD RS-IV)^18^, rating one-four (“never” is rated as 1, “occasionally” is 2; “often” is 3; “always” is 4). This scale contains nine inattentive and nine hyperactive/impulsive symptoms of ADHD described in the DSM-IV. The higher the scores were, the more serious the ADHD symptoms were.

Besides, impulsivity–hyperactivity and hyperactivity index in the Conners’ Parent Rating Scale (CPRS)^19^ were used to assess the hyperactive/impulsive symptoms in child participants with ADHD. The CPRS is a widely used instrument for screening and evaluating ADHD-related symptoms as well as other behavioral problems frequently associated with ADHD in children. It contains 48 items and can be divided into six factors: conduct problems, learning problems, psychosomatic problems, impulsivity–hyperactivity, anxiety, and hyperactivity index. The parents rate each item using a 4-point Likert-type scale (“never/seldom” is rated as 0, “sometimes” is 1; “quite often” is 2, and “very often” is 3). The higher the score, the more severe the corresponding problem is.

This study was approved by the Research Ethics Review Board of Peking University Sixth Hospital and Beijing Normal University. All subjects provided written informed consent and were fully informed about the research.

### Resting-state functional connectivity analysis

Specific parameters for scanning were shown in the supplement. Two datasets were both preprocessed using the Data Processing Assistant for Resting-State fMRI^20^ (DPARSFA, http://rfmri.org/DPARSF). The first 10 volumes were discarded to allow for magnetization equilibrium. Subsequent data preprocessing included slice timing correction, head motion correction, spatial normalization to the MNI template, resampling to 3 × 3 × 3 mm^3^, smoothing using a 4 mm Gaussian kernel, temporal band-pass filtering (0.01 Hz to 0.1 Hz), nuisance signal regression (including six head motion parameters, white matter, cerebrospinal fluid, and global signals), and head motion scrubbing (the mean frame-wise displacement, as described by Jenkinson et al.^21^). The registered fMRI volumes with the MNI template were divided into 273 regions according to the Brainnetome Atlas^22^ incorporating 210 cortical, 36 subcortical, and 27 cerebellar regions.

Regional mean time series were obtained for each by averaging the fMRI time series over all voxels in each of the 273 regions. Pearson correlation coefficients between pairs of node time courses were calculated and normalized to *z* score using Fisher transformation, resulting in a 273 × 273 symmetric connectivity matrix for each subject. Removing 273 diagonal elements, we extracted the upper triangle elements of the functional connectivity matrix as prediction features, i.e., the feature space for prediction was spanned by the (273 × 272)/2 = 37,128 dimensional feature vectors. In this study, FC features were described as inter-network FCs and intra-network FCs. According to the study of Yeo and his colleagues^23^, brain regions can be grouped into seven functional networks for visualization: visual network (VN), somatomotor network (SMN), dorsal attention network (dATN), ventral attention network (vATN), limbic network (LN), frontoparietal network (FPN), and default mode network (DMN).

### Ensemble feature selection algorithm

To investigate diagnostic features between ADHD and HCs, FS_RIEL was proposed to extract most-discriminative features from high-dimensional FCs and improve the result interpretability by estimating the relative importance of features, which refers to the degree of features (i.e., FC node) contribute to classification. Five different algorithms including extreme gradient boosting^24^, randomized decision trees (a.k.a. ExtraTrees^25^), Random Forest^26^, AdaBoost^27^, and Gradient Boosting^28^ were employed to select features with the top 2% relative importance from different models respectively, which were assembled into a feature pool without any repetition. Then, all pooled features were fed into a linear support vector machine with a forward-backward searching strategy (SVM-FoBa)^29^, obtaining a more refined feature subspace. After that, the label of each subject was calculated by majority voting of the 5 base classifiers used in training. We adopted nested 3-fold cross-validation on the whole training and validation set. Please see more details in Supplementary Figs. S1 and S2 on method flowchart. To verify the validity of features we selected as well as the classification performance, we also compared the proposed model with four traditional methods including Lasso^30^, ElasticNet^31^, Fisher-Score^32^, and Trace_Ratio^33^ (Kolmogorov–Smimov test) on the classification accuracy, sensitivity, specificity, and feature dimension.

### Within-cohort classification and cross-cohort prediction

We first performed the within-cohort classification as mentioned above to extract FCs that can discriminate ADHD from age-matched controls within child dataset and adult dataset, respectively. Then the identified ADHD-discriminative FCs were compared between child and adult cohorts, resulting in the shared and age-specific FCs between ADHD_child_ and ADHD_adult_. Next, the correlation between ADHD symptoms and the selected shared and distinct FC patterns were further calculated, deriving out some interesting observations.

**Fig. 1:**
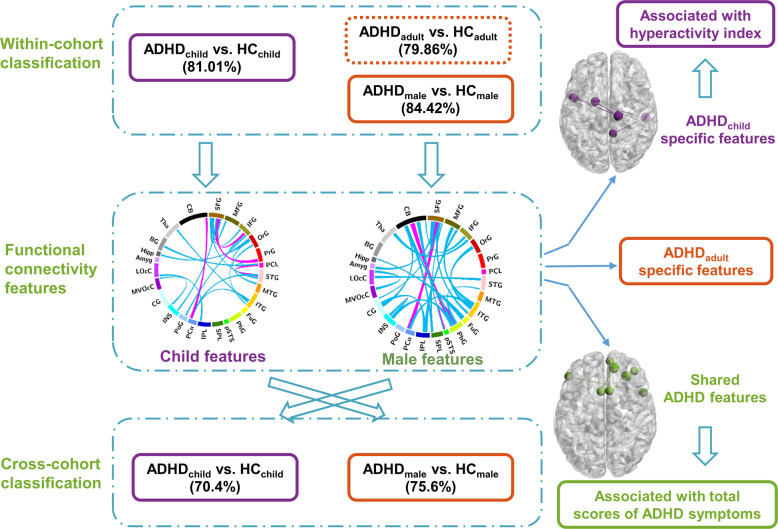
Flowchart of the whole study design. We first performed the within-cohort classification to extract shared and distinct FCs between ADHD_child_ and ADHD_adult_, and then the correlation between ADHD symptoms and the identified FC patterns were further calculated. Moreover, to investigate the stability/continuity in diagnosing of ADHD from childhood to adulthood, a cross-cohort prediction using the identified FCs in discovery cohort were performed. ADHD attention-deficit/hyperactivity disorder, HC healthy control.

Moreover, to investigate the stability/continuity in diagnosing of ADHD from childhood to adulthood, a cross-cohort prediction using the identified FCs in discovery cohort were performed. Namely, classifying ADHD_child_ from HC_child_ using features extracted from the ADHD_adult_ cohort classification, and vice versa. Since the participants in child dataset were all boys, hence only male participants (74 male ADHD adult patients and 43 age-matched HCs) were selected for the cross-cohort prediction. The flowchart of the whole study design was shown in Fig. 1.

**Fig. 2:**
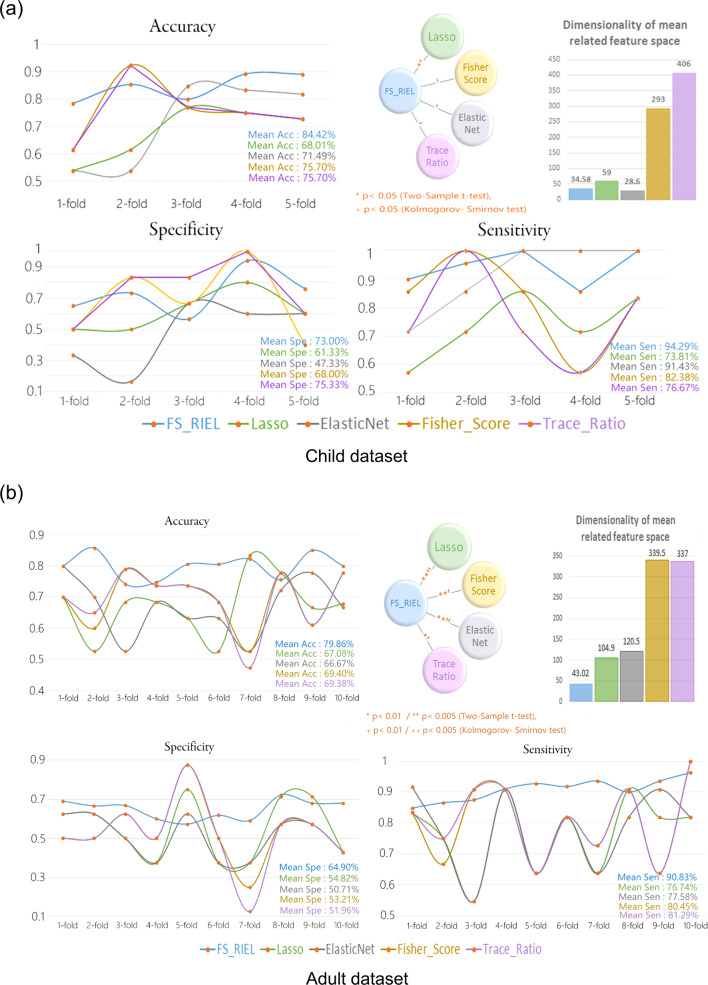
The performance of our proposed model in child and adult datasets. Five-fold/ten-fold cross-validation in (**a**)/(**b**) were used to validate FS_RIEL’s performance (accuracy, specificity, and sensitivity). Looping five/ten times, the averaged classifying accuracy, specificity, and sensitivity are regarded as the whole classification performance. The accuracy with FS_RIEL was significantly higher than those with the other four popular methods. The dimensionality of mean related feature space is also shown. FS_RIEL feature selection method based on relative importance and ensemble learning, Acc accuracy, Spe specificity, Sen sensitivity.

In order to make full use of the data set, a k-fold cross-validation strategy was used to estimate the performance of discriminating ADHD from HCs (accuracy, sensitivity, and specificity). The steps of k-fold cross-validation are as follows: the data set is divided into k parts and taken turns using k-1 parts as a training set and the remaining one part as a test set. After looping k times, all subjects were guaranteed to be used in test set independently, and finally, the averaged classifying accuracy of k times is regarded as the whole classification accuracy^34^. Specific, every looping was repeated 10 times in this study to ensure the stability of the results. K-fold cross validation is a resample procedure usually used to evaluate the classification performance of the model on a limited dataset, which can reduce the over fitting to a certain extent. After mean value across all k trials is computed, this scheme matters less which part of subjects are used as training set and which are used as testing set. Further, it can also improve the stability of the results and obtain as much effective information as possible from the limited data. Here, 5-fold cross-validation was used in child dataset (Fig. 2a) and 10-fold cross-validation was used to in adult dataset (Fig. 2b), as the number of children was relatively small.

## Results

### Demographic and clinical characteristics

Demographics and clinical characteristics of the ADHD patients and HCs were shown in supplementary Tables S1–S3. As expected, age, sex, and IQ were not significantly different between ADHD patients and HCs, except for lower IQ in ADHD_child_ than HCs. Total symptoms, inattentive, and hyperactive/impulsive symptoms rated with the ADHD RS-IV were significantly higher in participants with ADHD than HCs. Details about ADHD subtypes, comorbidities, and medication history were shown in Supplementary Tables S1–S3. ADHD patients still taking methylphenidate hydrochloride or tomoxetine hydrochloride were required to undergo a washout period of at least 5 days before the MRI scan. The mean half-life of methylphenidate hydrochloride was ~3.6 h, while the half-life of tomoxetine hydrochloride is ~5 h for extensive metabolizers and about 22 h for poor metabolizers. After five half-lives (it is 110 h for poor metabolizers with tomoxetine hydrochloride), all drugs in the body were eliminated. In order to facilitate the implementation, take the minimum integer days, that is, 5 days.

### Within-cohort classification

As shown in Fig. 2a, the accuracy, sensitivity, and specificity of discriminating ADHD_child_ from HC_child_ were 84.42%, 94.29%, and 73.00%, respectively. The classification accuracy of the child data with the proposed model was significantly higher than those with Lasso (*p* = 0.004), ElasticNet (*p* = 0.031), Fisher-score (*p* = 0.036), and Trace_Ratio (*p* = 0.036). Except the dimensionality of mean related feature space with ElasticNet in child dataset, the dimensionality of FS_RIEL was the lowest (see Fig. 2a).

The accuracy, sensitivity, and specificity of discriminating ADHD_adult_ from HC_adult_ (all adult participants, including males and females) with FS_RIEL were 79.86%, 90.83%, and 64.90%, respectively. The discriminating accuracy obtained by FS_RIEL was significantly higher than those obtained by Lasso (*p* = 0.003), ElasticNet (*p* = 0.001), Fisher-Score (*p* = 0.015), and Trace_Ratio (*p* = 0.015, see Fig. 2b). In addition, the feature dimensionality selected by FS_RIEL was lower than the other four methods above (see Fig. 2b). Further, if using only male adults, the accuracy, sensitivity, and specificity of ADHD-HC classification were 81.01%, 90.58%, and 64.55%, respectively, using FS_RIEL.

### The shared and distinct FCs between ADHD_child_ and ADHD_adult_

**Fig. 3:**
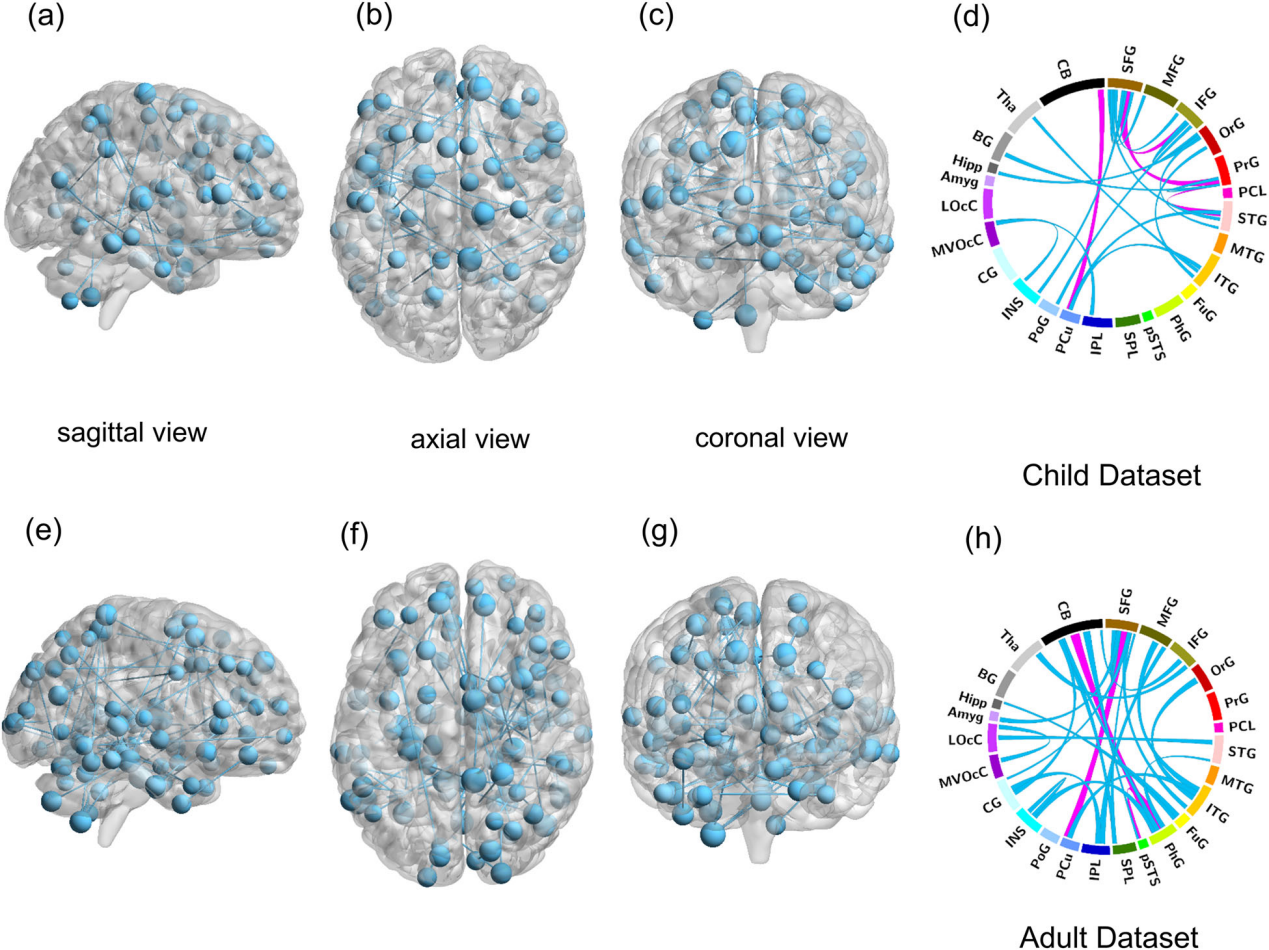
Functional connectivities in child and adult datasets. The blue and pink lines illustrate FCs extracted via our proposed algorithm. The most discriminating FCs in child dataset was shown in **a**–**d**. The most discriminating FCs in adult dataset was shown in **e**–**h**. Lines with more width denote more frequency used in a new space, and pink lines were top three FCs in **d** and **h**. FCs functional connectivities, ADHD attention-deficit/hyperactivity disorder.

We compared the selected FC features to explore the shared and distinct FCs of adults and children with ADHD in males because all participants were boys in the child dataset. Figure 3 showed the most important FCs (frequency of FCs occurring in all loops were ≥10 in within-cohort classification). The top four discriminating FCs to classify ADHD_child_ from HC_child_ were the following: ventromedial prefrontal cortex-precentral gyrus, cerebellum-precuneus, superior temporal gyrus-precentral gyrus, and connectivity within ventromedial prefrontal cortex (the frequency of the last two FC is the same). The top three discriminating FCs to classify ADHD_adult-_HC_adult_ (only including males) were as follows: superior temporal gyrus-fusiform gyrus, ventromedial prefrontal cortex-precuneus, and cerebellum-fusiform gyrus.

**Fig. 4:**
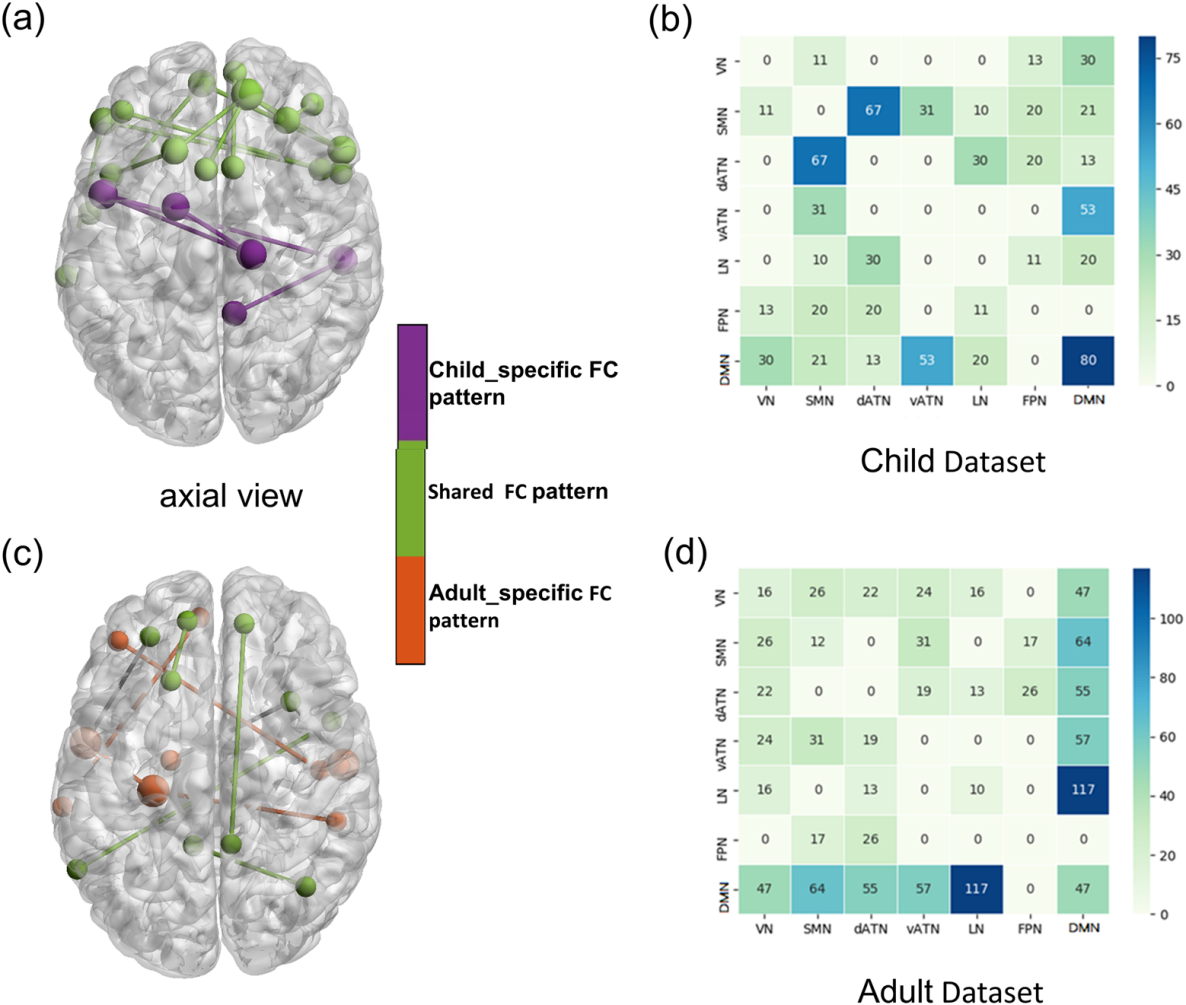
Intra- and inter-network connectivity of the most discriminating functional connectivities in child and adult datasets. Shared FC patterns lie in intra-network within DMN and inter-network between DMN and vATN between child dataset (green in **a**) and adult dataset (green in **c**). On the other side, the inter-network FC between SMN and dATN was uniquely impaired in ADHD_child_ (purple in **a**); whereas the aberrant inter-network FC between DMN and LN was indicated more adult-specific ADHD dysfunction (orangered in **c**). Intra- and inter-network connectivity of the most discriminating FCs in child and adult datasets were shown in **b** and **d**, respectively. VN visual network, SMN somatomotor network, dATN dorsal attention network, vATN ventral attention network, LN limbic network, FPN frontoparietal network, DMN default mode network, FCs functional connectivities.

The distribution of the most important FC nodes distributed within or among well-known brain networks, i.e., intra-network and inter-network was shown in Fig. 4. The most important intra-network FCs were located within the DMN, while the most important inter-network FCs were between DMN and vATN. This observation was common in both child and adult datasets.

As to the age-specific ADHD-discriminative FCs, the aberrant inter-network FC between SMN and dATN only exist in child dataset. While the aberrant inter-network FC between DMN and LN was less prominent in child than in adult dataset. Further analysis showed that all of the above FCs did not significantly differ between ADHD patients and HCs in both child and adult datasets.

### Association between functional connectivity and clinical symptoms

**Fig. 5:**
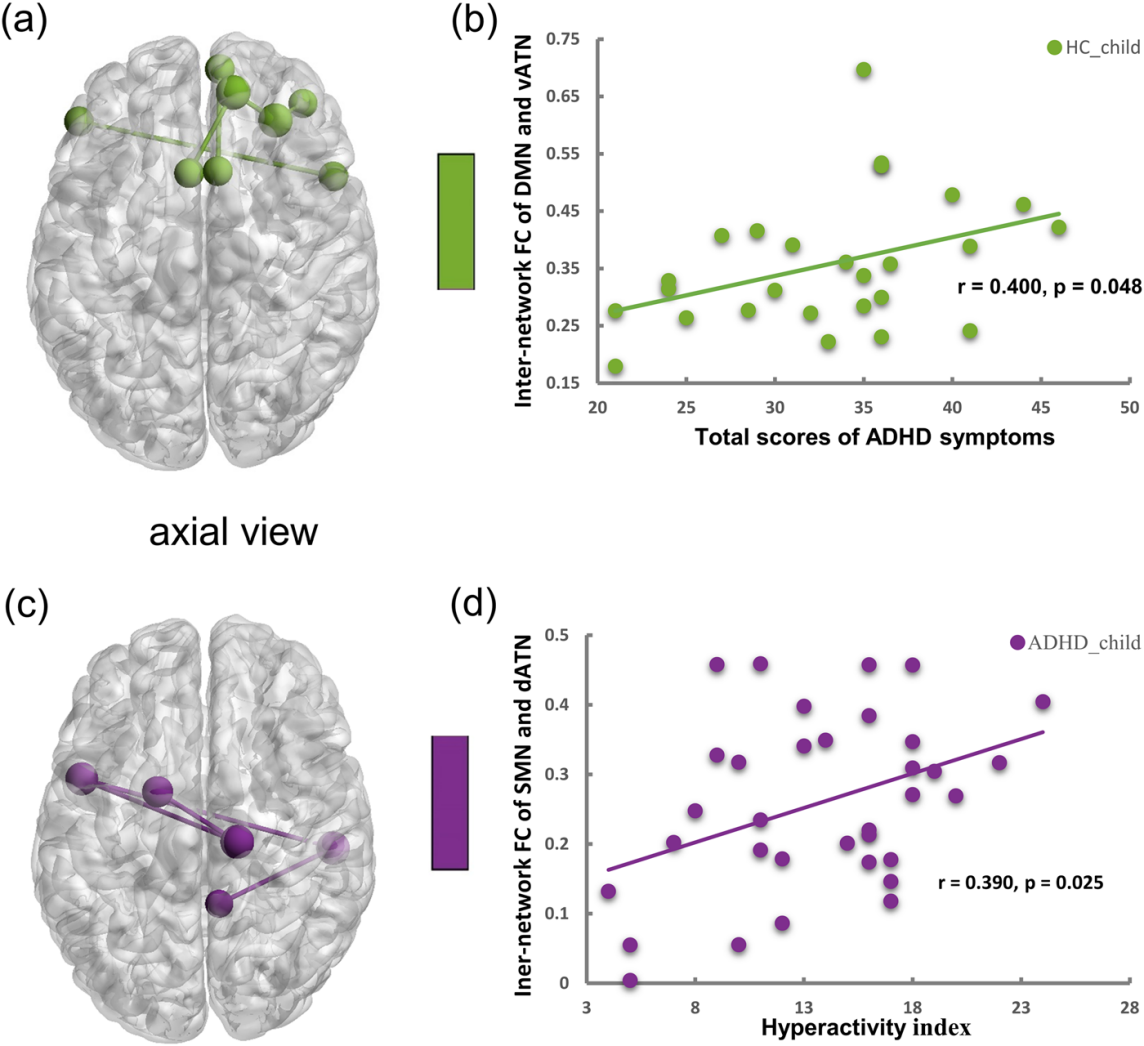
The correlation of inter-network functional connectivity and clinical symptoms. Inter-network FC between DMN and vATN (**a**) was significantly correlated with total scores of ADHD symptoms in HC_child_ group (**b**). Inter-network FC between SMN and dATN (**c**) was positively associated with hyperactivity index scores in ADHD_child_ group (**d**). FC functional connectivity, ADHD attention-deficit/hyperactivity disorder, HC healthy control, DMN default mode network, SMN somatomotor network, dATN dorsal attention network, vATN ventral attention network.

We calculated correlation with 5 subscales of ADHD symptoms. Interestingly, the inter-network FC between DMN and vATN showed a significant positive correlation with total scores of the ADHD RS-IV (*r* = 0.400, *p* = 0.048) in HC_child_ group, but not in ADHD_child_ group. Besides, the above variables have a positive correlation trend in adults (*r* = 0.191, *p* = 0.061, controlling for group). Moreover, inter-network FC between SMN and dATN was positively associated with hyperactivity index scores of the CPRS in ADHD_child_ group (*r* = 0.390, *p* = 0.025) (see Fig. 5). The other clinical variables were not significantly correlated with the identified FCs.

### Cross-cohort prediction

In addition to within cohort prediction, we are curious about whether and to what extent the identified FC features of the ADHD_child_ could predict ADHD_adult_, and vice versa. The accuracy, sensitivity, and specificity of discriminating ADHD_adult_ from HC_adult_ by directly using the features trained from ADHD_child_ dataset were 75.6%, 89.0%, and 53.0% respectively. In contrast, the accuracy, sensitivity, and specificity of classifying child dataset using the classifier trained on the ADHD_adult_ cohort were 70.4%, 80.0%, and 58.7% respectively (see supplementary Table S4).

## Discussion

In this work, we designed a dedicated imaging study by using both within-cohort classification and cross-cohort prediction based on two valuable ADHD cohorts in order to investigate shared and distinct FC patterns in ADHD_child_ and ADHD_adult_, and further investigated the pathophysiological continuity in ADHD from childhood to adulthood using the identified FCs. Besides the appreciable classification accuracy achieved (ADHD_child_: 84.4%, ADHD_adult_ 81.0%, and >70% for cross-cohort prediction), results further indicated that there are shared ADHD-discriminative FCs between children and adults, which lie in the intra-network within DMN and the inter-network between DMN and vATN, and is also positively correlated with total ADHD symptoms score. On the other side, the inter-network FC between SMN and dATN was uniquely impaired in ADHD_child_, positively correlated with hyperactivity index; whereas the aberrant inter-network FC between DMN and LN exhibited more adult-specific ADHD dysfunction. These results showed that shared and distinct patterns of FC were significantly related to symptomatic persistence and changes in children and adults with ADHD and further provided an additional evidence for the continuity of pathophysiology in ADHD. We identified both shared, and age-specific FC patterns impaired in ADHD and further investigated the cross-age predictability using the identified FCs. To the best of our knowledge, this is the first attempt to make a mutual prediction between children and adults with ADHD.

One of the most key issue in ADHD machine learning studies is reliability. Here, we will state the reliability of the results from the accuracies of the discrimination and the clinical significance of the identified FCs. Firstly, the classifying accuracy of the two cohorts are nearly or over 80%, both significantly higher than four popular machine learning method. Many previous studies only include one dataset, lack of independent datasets to verify the classifying performance^13,35,36^. One published study classified children with ADHD from HCs in different datasets. The classification accuracies in four datasets were 81.8%, 44.0%, 60.9%, and 64.7% respectively^14^. Though the classifying accuracy was relatively high in one dataset, the accuracies of the other three datasets were unsatisfactory with low reproducibility. The most critical deficiency in the above studies is the potential lack of generalization of the prediction models. This lack of testing of the models on independent validation sets, especially on different age ADHD patients whose neural mechanisms were not exactly the same as ADHD is a neurodevelopmental disorder is a major limitation. In this work, it included both children and adults with ADHD cohorts and reached a relatively high classifying accuracy. Secondly, the identified FCs are consistent not only with previous findings but also with symptom changes of ADHD observed in the clinics (details see the following discussion). The above results suggest that the findings are reliable and clinically meaningful in this work.

### Shared FC patterns in children and adults with ADHD

The DMN, mainly including medial prefrontal cortex, superior frontal gyrus, posterior cingulate cortex, precuneus cortex, involved in ADHD-linked symptoms including mind-wandering and attentional fluctuations, has been shown to performance abnormal inter-network and intra-network FCs in ADHD^37^, just consistent with our results that aberrant intra-network FC within DMN and inter-network FC between DMN and vATN were shared in ADHD_child_ and ADHD_adult_. In the past decade, aberrant FCs of the DMN have been the most frequently reported brain network in ADHD^38^. The default-mode interference hypothesis points out that attention deficit, at least in part, is due to the failure to fully and effectively transform from a baseline “default mode” to an active processing mode during cognitive tasks^39^. Consistent with this hypothesis, recurrent results indicated atypical brain FCs within the DMN in children and adults with ADHD^38^ and suggested potentially important roles of this network to the pathophysiology of ADHD.

Independent lines of studies suggested that attention deficits in ADHD were associated with altered intrinsic connectivity networks interrelationships. According to current theoretical models^39,40^, inattention in ADHD involves altered competitive balance between DMN and several task-positive networks, especially vATN. The DMN was involved in internally directed mentation, and task-positive networks were related to externally directed cognitive tasks^41^. The vATN (often called “salience network”) was a key task-positive network and thought to be responsible for regulating switching between externally focused and internally directed mentation^42^. Lapses of attention arose when inappropriate intrusion of DMN during externally focused tasks due to ineffective regulation by vATN^39^. Previous studies have also confirmed this view. A study showed altered resting state connectivity between DMN and vATN in children with ADHD^43^. Similarly, adults with ADHD showed aberrant resting FC within vATN and DMN^44^. Our results showed a significant positive correlation between vATN-DMN interconnections and total scores of ADHD symptoms in HC_child_ group. Such correlation of these variables was disrupted in ADHD_child_ group. It suggested that within a certain range, with the increase of ADHD symptom scores, FCs of vATN and DMN increased to maintain a normal level of subjects’ functions. However, when the score of symptoms exceeded a certain threshold, FCs between vATN and DMN lost this compensatory regulation, that is, with the increase of the score of symptoms, FCs did not make corresponding compensatory changes, leading to the occurrence of ADHD and dysfunctions.

### Distinct FC patterns in children and adults with ADHD

We found that aberrant inter-network FC between SMN and dATN was unique for children with ADHD. Long-held clinical observations reported that hyperactive and impulsive symptoms decreased with age^45^, aberrant FCs in SMN would be expected in children with ADHD given the salience of motoric hyperactivity. In line with this clinical observations, a meta-analysis across 55 fMRI studies in ADHD showed that hypoactivation in the SMN was less prominent in adults than in children^6^. The dATN whose core regions include intraparietal sulcus and the frontal eye field generates and maintains endogenous signals based on current targets and pre-existing information of possible emergencies, and sends out top-down signals to direct the processing of appropriate stimulus features^46^. The dATN is also involved in associating relevant stimuli with appropriate motor responses^46^. Thus aberrant inter-network FC between dATN and SMN might underpin impulsivity/hyperactivity and occur only in children with ADHD. Our results showed that dATN-SMN interconnections were significantly associated with hyperactivity index, not with impulsive/hyperactive scores assessed by both the ADHD RS-IV and the CPRS. It suggested that dATN-SMN interconnections might correlated with hyperactive behaviors, not with impulsive behaviors. This requires further study.

We also observed that aberrant inter-network FC between DMN and LN was less prominent in ADHD_child_ than in ADHD_adult_. Abnormal FCs of limbic system with the DMN might underlie the susceptibility to affective disorders in ADHD^47^. Limbic/affective network (which mainly includes amygdala, hippocampus, subgenual cingulate cortex, orbitofrontal cortex, and nucleus accumbens) is involved in emotional regulation and monitoring of the salience of motivational stimuli^48^. In addition, emotion regulation relies on the ability to direct attention towards or deflect attention away from emotional stimuli so as to maintain emotional homeostasis or maintain focus on a goal^49^. The DMN network (medial and ventrolateral prefrontal cortex) may support the abnormal allocation of attention to emotional stimuli and could thus be regarded as the major ‘top-down’ contributor to emotion dysregulation within ADHD^50^. Together with already evident attention deficits in ADHD, the deficits in the allocation of attentional resources in emotional stimuli would exacerbate emotion dysregulation. Emotion dysregulation is found in around 25–45% of children and between 30 and 70% of adults with ADHD^50^. Since affective disorders and emotion dysregulation were more prominent in adults with ADHD, this would be expected that aberrant inter-network FC between DMN and LN was more prominent in ADHD_adult_ than in ADHD_child_. Unfortunately, we did not collect scores on anxiety or depression symptoms.

Further analysis showed that all of the shared and distinct patterns of FC did not significantly differ between ADHD patients and HCs in both child and adult datasets. There were two possible explanations. The first possible reason was that the features were selected based on relative importance, namely the degree of these features to discriminating ADHD from HCs, rather than on significant difference between groups. The clinical significance of these features did not always mean that these features were significantly different between patients and healthy controls. More likely, the statistical power of simple linear comparisons was insufficient for high-dimensional and nonlinear FC features^12^. Whereas machine learning showed great advantages in exploring the core features of psychiatric disorder characterized with deficits in heterogeneous distributed systems^12,51^. Compared with four popular feature selection algorithms, our proposed method not only can classify ADHD from HCs more accurately but also can reduce original feature space into a much more refined subspace, suggesting the FC features extracted with our method are probably the more core deficits of ADHD. Besides, the accuracy was 75.6% for classification of adults with ADHD using features trained from the child dataset, and 70.4% for classification of children with ADHD using features trained from the adult dataset. This added an objective imaging evidence for the continuity of ADHD from childhood to adulthood.

### Limitations

There are some limitations to this study. We studied the shared and distinct patterns of FC in children and adults with ADHD. But all participants were males, and the results might not generalize to females with ADHD. Yet, a small proportion of patients had a history of medicine. ADHD patients were required to undergo a washout period of at least 5 days before the MRI scan to decrease the possible effects. In addition, specific frequency bands [i.e., slow-6 (<0.01 Hz), slow-5 (0.01–0.027 Hz), slow-4 (0.027–0.073 Hz), slow-3 (0.073–0.198 Hz), and slow-2 (0.198–0.25 Hz)] may contribute differentially to functional connectivity. Only conventional frequency band of 0.01–0.1 Hz was used in this study considering this frequency band was thought to be mainly linked to neuronal fluctuations^52^. It would be an interesting topic to focus on the common and distinct functional connectivity with different frequency bands in children and adults with ADHD in future.

### Implications

In conclusion, we demonstrated that aberrant intra-network FC within DMN and inter-network FC between DMN and vATN were shared in children and adults with ADHD, consistent with default-mode interference hypothesis. Moreover, the results provided evidence that aberrant inter-network FC between SMN and dATN was unique for children with ADHD, in line with that hyperactive and impulsive symptoms decreased with age in ADHD. Whereas aberrant inter-network FC between DMN and LN was indicated more adult-specific ADHD dysfunction. Our findings highlight both shared, and distinct patterns of FC were correlated to symptoms in children and adults with ADHD. These features could be further used to partly make a mutual prediction between children and adults with ADHD, suggesting that FCs may serve as a potential biomarker for ADHD diagnosis. This work may shed new light on the underlying mechanisms of ADHD and provide objective imaging evidence for symptomatic changes and pathophysiological continuity in ADHD from childhood to adulthood.

## Supplementary information

supplementary material

## References

[1] Biederman J (2012). Adult outcome of attention-deficit/hyperactivity disorder: a controlled 16-year follow-up study. J. Clin. Psychiatry.

[2] Simon V, Czobor P, Bálint S, Mészáros Á, Bitter I (2009). Prevalence and correlates of adult attention-deficit hyperactivity disorder: meta-analysis. Br. J. Psychiatry.

[3] Bos DJ (2017). Structural and functional connectivity in children and adolescents with and without attention deficit/hyperactivity disorder. J. Child Psychol. Psychiatry.

[4] de Lacy N, Kodish I, Rachakonda S, Calhoun V (2018). Novel in silico multivariate mapping of intrinsic and anticorrelated connectivity to neurocognitive functional maps supports the maturational hypothesis of ADHD. Hum. Brain Mapp..

[5] Rubia K, Alegria A, Brinson H (2014). Imaging the ADHD brain: disorder-specificity, medication effects and clinical translation. Expert Rev. Neurother..

[6] Cortese S (2012). Toward systems neuroscience of ADHD: a meta-analysis of 55 fMRI studies. Am. J. Psychiatry.

[7] Faraone SV, Biederman J (2016). Can attention-deficit/hyperactivity disorder onset occur in adulthood?. JAMA Psychiatry.

[8] Sergeant JA, Geurts H, Huijbregts S, Scheres A, Oosterlaan J (2003). The top and the bottom of ADHD: a neuropsychological perspective. Neurosci. Biobehav. Rev..

[9] Konrad K, Eickhoff SB (2010). Is the ADHD brain wired differently? A review on structural and functional connectivity in attention deficit hyperactivity disorder. Hum. Brain Mapp..

[10] Samea F (2019). Brain alterations in children/adolescents with ADHD revisited: A neuroimaging meta-analysis of 96 structural and functional studies. Neurosci. Biobehav. Rev..

[11] Braver TS (1997). A parametric study of prefrontal cortex involvement in human working memory. NeuroImage.

[12] Cohen JD (2017). Computational approaches to fMRI analysis. Nat. Neurosci..

[13] Sun H (2017). Psychoradiologic utility of MR imaging for diagnosis of attention deficit hyperactivity disorder: a radiomics analysis. Radiology.

[14] Riaz A, Asad M, Alonso E, Slabaugh G (2018). Fusion of fMRI and non-imaging data for ADHD classification. Comput. Med. Imaging Graph..

[15] Kaufman J (1997). Schedule for Affective Disorders and Schizophrenia for School-Age Children-Present and Lifetime Version (K-SADS-PL): initial reliability and validity data. J. Am. Acad. Child Adolesc. Psychiatry.

[16] Conners C, Erhardt D, Sparrow M (1999). Conner’s Adult ADHD Rating Scales: CAARS..

[17] First M, Spitzer R, Gibbon M, Williams J (1996). Structured Clinical Interview for DSM-IV ® Axis I Disorders (SCID-I), Clinician Version..

[18] DuPaul GT, Power TJ, Anastopoulos AD, Reid R (1998). ADHD Rating Scale-IV: Checklists, Norms, and Clinical Interpretation..

[19] Conners CK, G S, Jd P, Epstein JN (1998). The revised Conners’ Parent Rating Scale (CPRS-R): factor structure, reliability, and criterion validity. J. Abnorm. Child Psychol..

[20] Yan CG, Zang YF (2010). DPARSF: a MATLAB toolbox for “Pipeline” data analysis of resting-state fMRI. Front. Syst. Neurosci..

[21] Jenkinson M, Bannister P, Brady M, Smith S (2002). Improved optimization for the robust and accurate linear registration and motion correction of brain images. NeuroImage.

[22] Fan L (2016). The human brainnetome atlas: a new brain atlas based on connectional architecture. Cereb. Cortex.

[23] Yeo BT (2011). The organization of the human cerebral cortex estimated by intrinsic functional connectivity. J. Neurophysiol..

[24] Chen T., Guestrin C. XGBoost: A Scalable Tree Boosting System. In *Proc. 22nd ACM SIGKDD International Conference on Knowledge Discovery and Data Mining* (San Francisco, CA, USA, 2016).

[25] Geurts P, Ernst D, Wehenkel L (2006). Extremely randomized trees. Mach. Learn..

[26] Breiman L (2001). Random forests. Mach. Learn..

[27] Freund Y, Schapire R (2010). A decision-theoretic generalization of on-line learning and an application to boosting. J. Comput. Syst. Sci..

[28] Friedman JH (2001). Greedy function approximation: a gradient boosting machine. Ann. Stat..

[29] Jie NF (2015). Discriminating bipolar disorder from major depression based on SVM-FoBa: efficient feature selection with multimodal brain imaging data. IEEE Trans. Auton. Ment. Dev..

[30] Tibshirani RJ (1996). Regression shrinkage and selection via the LASSO. J. R. Stat. Soc. Ser. B: Methodol..

[31] Hui Z, Hastie T (2005). Regularization and variable selection via the elastic net. J. R. Stat. Soc. B.

[32] Jaakkola T., M D., Haussler D. Using the Fisher kernel method to detect remote protein homologies. *Proc. Int. Conf. Intell. Syst. Mol. Biol.* 149–158 (1999). 10786297

[33] Nie F., Xiang S., Jia Y., Zhang C., Yan S. Trace ratio criterion for feature selection. *Proceedings of the Twenty-Third AAAI Conference on Artificial Intelligence* (Chicago, IIIionis, USA, 2008).

[34] Triba MN (2015). PLS/OPLS models in metabolomics: the impact of permutation of dataset rows on the K-fold cross-validation quality parameters. Mol. Biosyst..

[35] Castellanos FX, Di Martino A, Craddock RC, Mehta AD, Milham MP (2013). Clinical applications of the functional connectome. NeuroImage.

[36] Qureshi MNI, Oh J, Min B, Jo HJ, Lee B (2017). Multi-modal, multi-measure, and multi-class discrimination of ADHD with hierarchical feature extraction and extreme learning machine using structural and functional brain MRI. Front. Hum. Neurosci..

[37] Kucyi A, Hove MJ, Biederman J, Van Dijk KR, Valera EM (2015). Disrupted functional connectivity of cerebellar default network areas in attention-deficit/hyperactivity disorder. Hum. Brain Mapp..

[38] Fair DA (2010). Atypical default network connectivity in youth with attention-deficit/hyperactivity disorder. Biol. Psychiatry.

[39] Sonuga-Barke EJS, Castellanos FX (2007). Spontaneous attentional fluctuations in impaired states and pathological conditions: a neurobiological hypothesis. Neurosci. Biobehav. Rev..

[40] Castellanos FX, Proal E (2012). Large-scale brain systems in ADHD: beyond the prefrontal-striatal model. Trends Cogn. Sci..

[41] Buckner RL, Andrews-Hanna JR, Schacter DL (2008). The brain’s default network. Ann. N. Y. Acad. Sci..

[42] Menon V (2011). Large-scale brain networks and psychopathology: a unifying triple network model. Trends Cogn. Sci..

[43] Sripada CS, Kessler D, Angstadt M (2014). Lag in maturation of the brain’s intrinsic functional architecture in attention-deficit/hyperactivity disorder. Proc. Natl Acad. Sci. USA.

[44] McCarthy H (2013). Attention network hypoconnectivity with default and affective network hyperconnectivity in adults diagnosed with attention-deficit/hyperactivity disorder in childhood. JAMA Psychiatry.

[45] Faraone SV, Biederman J, Mick E (2005). The age-dependent decline of attention deficit hyperactivity disorder: a meta-analysis of follow-up studies. Psychol. Med..

[46] Corbetta M, Patel G, Shulman GL (2008). The reorienting system of the human brain: from environment to theory of mind. Neuron.

[47] Kim SM (2015). Affective network and default mode network in depressive adolescents with disruptive behaviors. Neuropsychiatr. Dis. Treat..

[48] Sheline YI, Price JL, Yan Z, Mintun MA (2010). Resting-state functional MRI in depression unmasks increased connectivity between networks via the dorsal nexus. Proc. Natl Acad. Sci. USA.

[49] Gross J (1998). The emerging field of emotion regulation: an integrative review. Rev. Gen. Psychol..

[50] Shaw P, Stringaris A, Nigg J, Leibenluft E (2014). Emotion dysregulation in attention deficit hyperactivity disorder. Am. J. Psychiatry.

[51] Pereira F, Mitchell T, Botvinick M (2009). Machine learning classifiers and fMRI: a tutorial overview. Neuroimage.

[52] Balduzzi D, Riedner BA, Tononi G (2008). A BOLD window into brain waves. Proc. Natl Acad. Sci. USA.

