# Supplementary material for: Shared and distinct resting functional connectivity in children and adults with attention-deficit/hyperactivity disorder
Source: Transl Psychiatry. 2020 Feb 12;10:65. doi: 10.1038/s41398-020-0740-y (PMC7026417; doi:10.1038/s41398-020-0740-y)
Supplement: Supplementary file 1 — supplementary material [file 41398_2020_740_MOESM1_ESM.docx]

Supplementary Information

**Shared and distinct resting functional connectivity in children and adults with attention-deficit/hyperactivity disorder**

**fMRI Data Acquisition and parameters for scanning**

fMRI data were acquired on a Siemens Trio 3T scanner (Siemens, Erlangen, Germany) at the Imaging Center for Brain Research at Beijing Normal University, and on a GE Signa 3T Horizon HDx system (General Electric, Milwaukee, WI) at the Centre for Neuroimaging Sciences, Peking University Sixth Hospital. Participants were required to lie in the supine position and remain still and relaxed with their eyes closed but not falling asleep during the resting-state fMRI scanning. An echo-planar imaging sequence with the following parameters were applied on the Siemens scanner: repetition time (TR) = 2,000 ms, echo time (TE) = 30 ms, flip angle = 90°, thickness/skip = 3.5/0.7 mm, matrix = 64 × 64, field of view (FOV) = 200 mm × 200 mm, 33 axial slices, and 240 volumes. High-resolution T1-weighted anatomical images were recorded with the following parameters: TR = 2,530 ms, TE = 3.39 ms, inversion time=1,100 ms, flip angle = 7°, 128 slices, slice thickness = 1.33 mm, FOV=256 mm× 256 mm, and matrix = 256 × 256. The parameters on the GE scanner were: TR = 2,000 ms, TE = 30 ms, flip angle = 90°, matrix = 64 × 64, FOV = 220 mm × 220 mm, 43 axial slices, slice thickness = 3.2 mm, slice gap = 0 mm in resting-state fMRI. The following parameters were in T1 on GE scanner: TR =6.7 ms, TE = Min Full, flip angle = 8°, 180 slices, slice thickness = 1.0 mm, slice gap = 0 mm, FOV = 256 mm × 256 mm, and matrix = 256 × 256.

**TABLES**

**Table S1.** **Demographic and Clinical characteristics for child dataset**

| **Characteristics** | **ADHD (N=34)** | **HC (N=28)** | **t** | ***p*** |
| --- | --- | --- | --- | --- |
| Age (years), mean (SD)  IQ, mean (SD)  Verbal IQ  Performance IQ | 10.49 (1.84)  108.41 (16.36)  98.85 (16.56) | 10.29 (1.67)  124.57 (12.47)  112.71 (16.41) | 0.446  -4.297  -3.293 | 0.657  <0.001  0.002 |
| Total IQ | 104.44 (15.66) | 121.25 (14.40) | -4.360 | <0.001 |
| ADHD RS-IV, mean (MD) |  |  |  |  |
| Total score | 50.16 (7.08) | 32.96 (6.72) | 9.464 | <0.001 |
| Inattentive score | 28.06 (3.65) | 17.23 (3.92) | 10.956 | <0.001 |
| HI score | 22.09 (5.80) | 15.73 (3.93) | 4.787 | <0.001 |
| CPRS, mean (MD) |  |  |  |  |
| HI score | 5.32 (2.74) |  |  |  |
| Hyperactivity index score | 13.74 (4.93) |  |  |  |
| ADHD Subtype (n) |  |  |  |  |
| Combined | 18 |  |  |  |
| Inattentive | 16 |  |  |  |
| Comorbidities (n) |  |  |  |  |
| ODD | 9 |  |  |  |
| tic disorder | 4 |  |  |  |
| Medication (n) |  |  |  |  |
| Previous MPH/ATX | 3 |  |  |  |

**Note:** All participants in child dataset were boys. ADHD: attention-deficit/hyperactivity disorder; HCs: healthy controls, IQ: intelligence quotient; ADHD RS-IV: ADHD Rating Scale-IV; HI: hyperactive/impulsive; CPRS： Conners' Parent Rating Scale; ODD: oppositional defiant disorder; MPH: methylphenidate; ATX: atomoxetine. Previous MPH/ATX means ADHD patients have stopped the treatment for a long time (> 1years).

**Table S2.** **Demographic and Clinical characteristics for adult dataset**

| **Characteristics** | **ADHD (N=112)** | **HCs (N=77)** | **t/χ^2^** | ***p*** |
| --- | --- | --- | --- | --- |
| Age(years), mean (SD) | 25.99 (0.46) | 26.04 (0.45) | 0.075 | 0.941 |
| Male/female (n) | 75/37 | 43/34 | 2.406^a^ | 0.129 |
| IQ, mean (SD) |  |  |  |  |
| Verbal IQ  Performance IQ | 120.21 (9.86)  117.99 (9.27) | 120.90 (8.50)  118.84 (7.61) | -0.416  -0.552 | 0.678  0.582 |
| Total IQ | 120.39 (9.04) | 121.56 (7.67) | -0.843 | 0.400 |
| ADHD RS-IV, mean (MD) |  |  |  |  |
| Total score | 45.47 (7.87) | 25.03 (5.80) | 17.610 | <0.001 |
| Inattentive score | 26.84 (4.18) | 12.98 (3.35) | 21.952 | <0.001 |
| HI score | 19.13 (5.07) | 12.04 (2.89) | 10.020 | <0.001 |
| ADHD Subtype (n) |  |  |  |  |
| Combined | 23 |  |  |  |
| Inattentive | 89 |  |  |  |
| Comorbidities (n) |  |  |  |  |
| Previous MDD | 18 |  |  |  |
| Anxiety disorder | 2 |  |  |  |
| Dysthymic disorder | 3 |  |  |  |
| Medication (n) |  |  |  |  |
| Previous MPH/ATX | 5 |  |  |  |
| Current MPH/ATX | 6 |  |  |  |

**Note:** ADHD: attention-deficit/hyperactivity disorder; HCs: healthy controls, IQ: intelligence quotient; ADHD RS-IV: ADHD Rating Scale-IV; HI: hyperactive/impulsive; MDD: major depressive disorder; MPH: methylphenidate; ATX: atomoxetine. Previous MPH/ATX means ADHD patients have stopped the treatment for a long time (> 1years).

^a^ means χ^2^ value.

**Table S3.** **Demographic and Clinical characteristics for male adults**

| **Characteristics** | **ADHD (N=74)** | **HC (N=43)** | **t** | ***p*** |
| --- | --- | --- | --- | --- |
| Age (years), mean (SD)  IQ, mean (SD)  Verbal IQ  Performance IQ | 26.02 (4.83)  119.91 (9.73)  116.68 (8.90) | 25.59 (3.18)  122.29 (8.57)  119.94 (7.97) | 0.519  -1.140  -1.169 | 0.605  0.257  0.093 |
| Total IQ | 119.83 (9.24) | 123.00 (6.88) | -1.834 | 0.070 |
| ADHD RS-IV, mean (MD) |  |  |  |  |
| Total score | 46.22 (8.38) | 24.19 (6.39) | 13.678 | <0.001 |
| Inattentive score | 27.27 (4.15) | 12.53 (3.48) | 18.037 | <0.001 |
| HI score | 19.70 (5.55) | 11.67 (3.32) | 7.919 | <0.001 |
| ADHD Subtype (n) |  |  |  |  |
| Combined | 13 |  |  |  |
| Inattentive | 61 |  |  |  |
| Comorbidities (n) |  |  |  |  |
| Previous MDD | 7 |  |  |  |
| Anxiety disorder | 1 |  |  |  |
| Dysthymic disorder | 2 |  |  |  |
| Medication (n) |  |  |  |  |
| Previous MPH/ATX | 5 |  |  |  |
| Current MPH/ATX | 5 |  |  |  |

**Note:** ADHD: attention-deficit/hyperactivity disorder; HCs: healthy controls, IQ: intelligence quotient; ADHD RS-IV: ADHD Rating Scale-IV; HI: hyperactive/impulsive; BRIEF-A: Behavior Rating Inventory Executive Function-Adult Version; MDD: major depressive disorder; MPH: methylphenidate; ATX: atomoxetine. Previous MPH/ATX means ADHD patients have stopped the treatment for a long time (> 1year).

**Table S4. The performance of a mutual prediction between child and adult dataset**

| **Variables** | **Accuracy** | **Specificity** | **Sensitivity** |
| --- | --- | --- | --- |
| **Child to adult** | 75.6% | 89.1% | 53.0% |
| **Adult to child** | 70.4% | 80.0% | 58.7% |

**Note:** Child to adult referred to the discrimination of adult patients with ADHD from healthy controls with features extracting from child dataset. Adult to child referred to the discrimination of child patients with ADHD from healthy controls with features extracting from adult dataset. ADHD: attention-deficit/hyperactivity disorder.

**FIGURES**

**
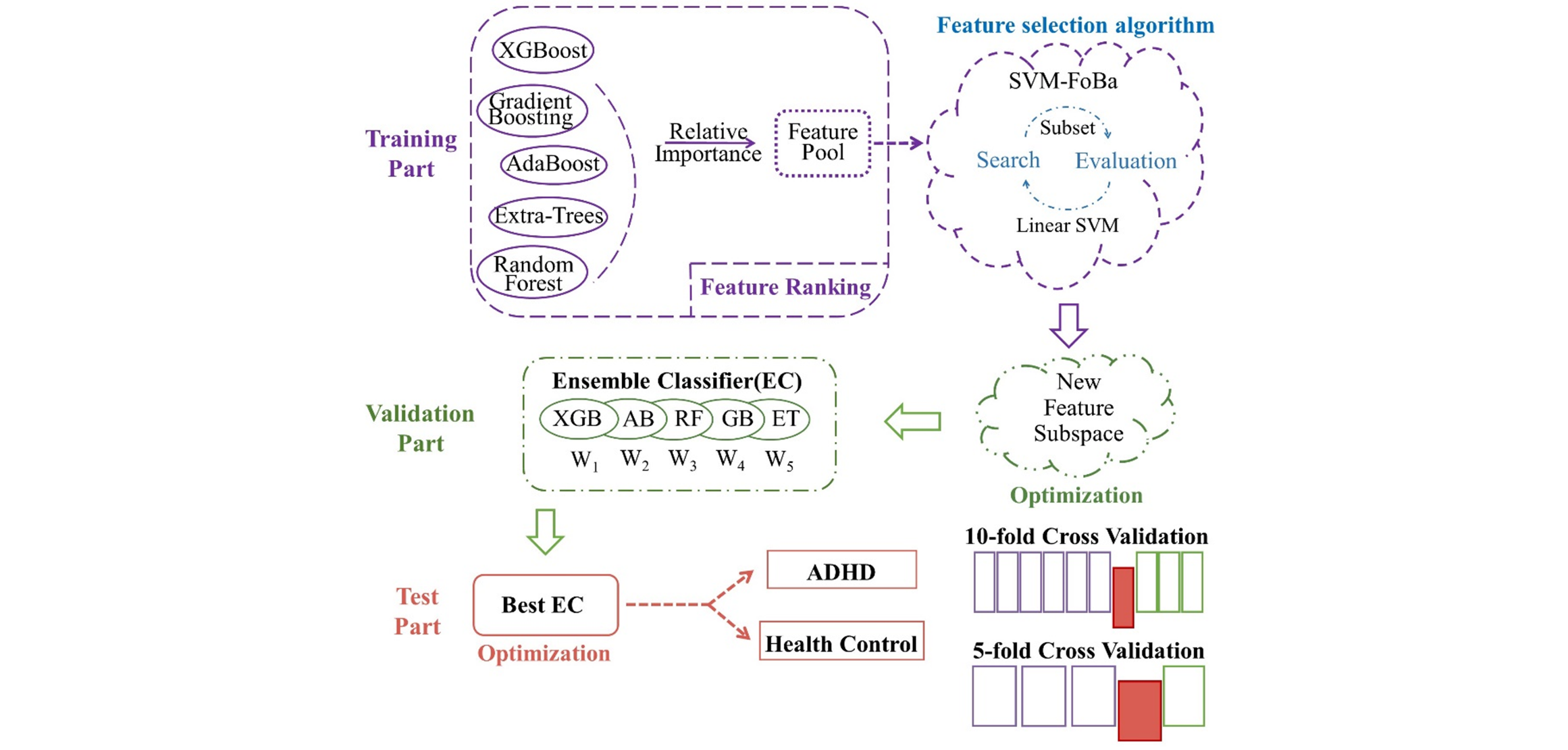
**

**Fig. S1** **Flowchart of our proposed feature selection method based on relative importance and ensemble learning algorithm.** Five different ensemble learning strategies are used for all FC features ranking based on relative importance. After top 2% features reserved, SVM-FoBa (see **Fig. S2**) is employed to generate new feature subspace. Then related weights with those used algorithms are learned under best performance on the validation set. The best ensemble classifier will be the discriminant criterion for test data. Note: ADHD: attention-deficit/hyperactivity disorder; XGBoost (XGB): extreme gradient boosting; AB: AdaBoost; RF: Random Forest; GB: Gradient Boosting; ET: Extra-Trees; SVM-FoBa: support vector machine with a forward-backward searching strategy.


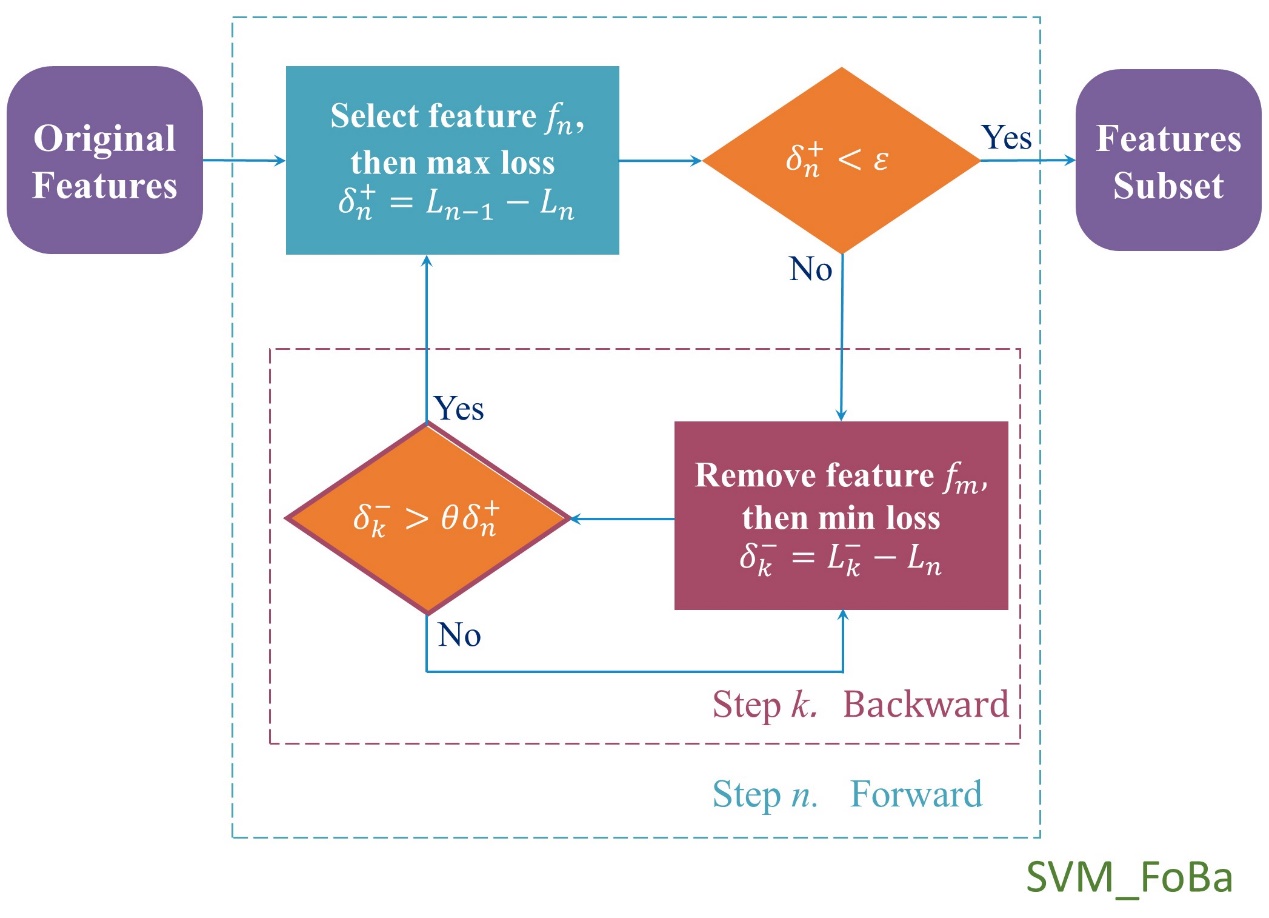


**Fig. S2 The support vector machine with a forward-backward searching strategy.** Each Forward step, one feature is added to the current set ***F*** in order to aggressively reduce the loss function ***L***; Backward elimination, features are removed one at a time so that the negative impact on performance shall be kept minimal. The details on support vector machine with a forward-backward searching strategy (SVM_FoBa) see Jie et al. (2015)^1^.

**REFERENCE**

1. Jie NF, et al. Discriminating Bipolar Disorder From Major Depression Based on SVM-FoBa: Efficient Feature Selection With Multimodal Brain Imaging Data. *IEEE Trans Auton Ment Dev* 2015;**7**:320-331.
